# First Molecular Identification of *Cotylophoron cotylophorum* in Ecuador and Its Phylogenetic Relationship with *Fasciola hepatica*

**DOI:** 10.3390/pathogens14070659

**Published:** 2025-07-04

**Authors:** Geanella Barragán-López, Fausto Bedoya-Páez, María Lugo-Almarza, Carolina Fonseca-Restrepo, Francisco Angulo-Cubillán, Edison J. Romero, Jacobus H. de Waard, Armando Reyna-Bello

**Affiliations:** 1 Grupo de Investigación en Sanidad Animal y Humana (GISAH), Departamento de Ciencias de la Vida y la Agricultura, Carrera de Biotecnología, Universidad de las Fuerzas Armadas ESPE, Sangolquí 171103, Ecuador; geanellabarragan@gmail.com (G.B.-L.); fvbedoya@espe.edu.ec (F.B.-P.); 2 Clínica Veterinaria del Pacífico, Santo Domingo 230101, Ecuador; mafer.lugo.almarza@gmail.com; 3 Carrera de Medicina Veterinaria, Facultad de Ciencias Veterinarias, Universidad Técnica de Manabí, Lodana 131320, Ecuador; carolina.fonseca@utm.edu.ec (C.F.-R.); francisco.angulo@utm.edu.ec (F.A.-C.); 4 Departamento de Ciencias de La Vida y la Agricultura, Universidad de las Fuerzas Armadas ESPE, Santo Domingo 230118, Ecuador; ejromero1@espe.edu.ec; 5 One Health Research Group, Facultad de Medicina Veterinaria, Universidad de las Américas, Quito 170125, Ecuador; jacobus.dewaard@udla.edu.ec

**Keywords:** *Cotylophoron cotylophorum*, Paramphistomum, fasciola hepatica, rumen flukes, liver flukes

## Abstract

Trematode infections caused by Fasciolidae and Paramphistomidae remain widespread in livestock, resulting in substantial economic losses. The two distinct fluke families are difficult to distinguish morphologically, and molecular identification provides the most reliable means of accurate diagnosis. In Ecuador, however, molecular data on these parasites are scarce. In this study, we collected trematodes from cattle rumen and bile ducts, molecularly identified them, and assessed their phylogenetic relationship to *Fasciola hepatica* to determine their introduction pathways into South America. Genomic DNA was extracted, and PCR was used to amplify the ITS2 (~500 bp) and COXI (~266 bp) regions; all amplicons were Sanger-sequenced. Phylogenetic trees for both markers were constructed using a Maximum Likelihood approach with 1000 bootstrap replicates in CIPRES v3.3. The rumen fluke exhibited 99% ITS2 and COXI similarity to an Indian *Cotylophoron cotylophorum* strain, while the bile-duct fluke showed 99% ITS2 and 100% COXI similarity to *F. hepatica* isolates from Australia and Nigeria, respectively. Distinct single-nucleotide polymorphisms (SNPs) in the ITS2 chromatograms suggest a diploid genome structure in both trematode species. This is the first report of *C. cotylophorum* in Ecuador, and its presence may be linked to the late 19th-century introduction of Zebu cattle (*Bos taurus indicus*) from India.

## 1. Introduction

Trematodiasis is a parasitic disease caused by flattened, non-segmented helminths (trematodes) that infect both animals and humans, negatively impacting health and welfare. In Ecuador, livestock farming is a key economic sector, so any disease affecting productivity can have broad socioeconomic consequences. Consequently, the diagnosis and control of parasitic infections are of vital importance [1].

In neighboring countries, two species of amphistome (rumen flukes) from the family Paramphistomidae have been reported in cattle: *Cotylophoron cotylophorum* in Colombia [2] and Venezuela [3] and *Calicophorum microbothrioides* in Peru [4]. In addition to their effects on livestock, paramphistomes have also been reported in humans, highlighting their zoonotic potential [5]. However, no records of Paramphistomidae species have been published in indexed journals from Ecuador.

*Fasciola hepatica* is another trematode of major veterinary and public health relevance, causing liver damage that compromises animal health and productivity [6]. This parasite has been previously reported in Ecuador [7], and its inclusion in the present study is relevant given that its life cycle closely resembles that of paramphistomes, including the use of the same intermediate snail hosts [3,8]. The presence and characterization of these intermediate hosts have also been described in Ecuador [9].

Molecular techniques are powerful tools for the precise identification of parasitic species, which is essential for implementing effective control strategies. Additionally, sequencing and comparing DNA from different regions allows researchers to trace the geographic origin, distribution, and relatedness of parasitic populations. The use of ITS2 and COXI gene markers has proven effective for molecular identification and phylogenetic analyses of trematodes, enabling the assessment of evolutionary relationships among specimens from various regions [10,11,12].

Based on this background, the present study aimed to molecularly identify an amphistome and liver fluke specimen found in Ecuadorian cattle and assess the phylogenetic relationship between the species.

## 2. Materials and Methods

### 2.1. Sample Collection

**Figure 1:**
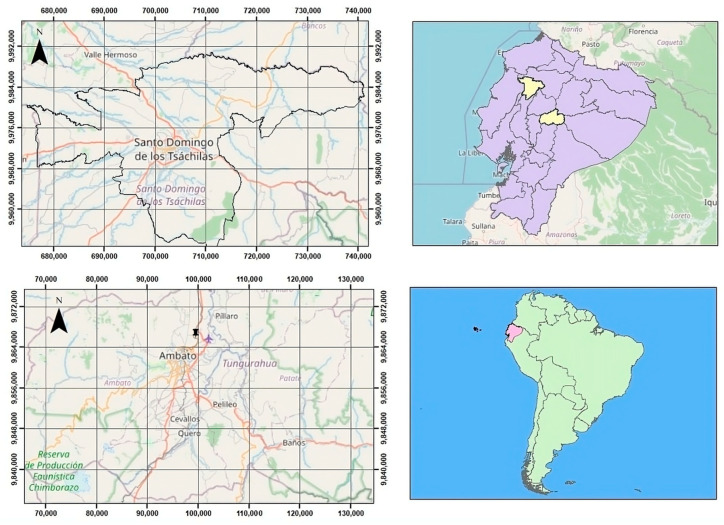
Geographical localization of the studied parasites in Ecuador. *Paramphistomidae* specimens were recovered in Santo Domingo province (top left panel), while *Fasciola hepatica* was collected from a slaughterhouse in Tungurahua province (bottom left panel). The provinces where samples were obtained are highlighted in pale yellow (upper right panel), and Ecuador’s position within South America is shown in green (lower right panel). Maps were created with QGIS (version 3.42), a free and open-source Geographic Information System (GIS) software.

The study was conducted at two locations in Ecuador (Figure 1). Twelve *Amphistomes* specimens were collected from the ruminal mucosa of a female bovine during a postmortem examination in the city of Santo Domingo. Based on their anatomical location and macroscopic features, the parasites were preliminarily identified as members of the family *Paramphistomidae*. Meanwhile, nine *Fasciola* specimens were obtained from the bile ducts of a bovine slaughtered at the Municipal Slaughterhouse in Ambato. Both trematode species were thoroughly rinsed with saline solution to eliminate host tissue residues, preserved in 70% ethanol, and transported to the “Universidad de las Fuerzas Armadas–ESPE”, where they were stored at −20 °C until further analysis.

### 2.2. DNA Extraction and Polymerase Chain Reaction (PCR)

DNA was extracted from both trematode species using the GeneJET Genomic DNA Purification Kit (Thermo Fisher Scientific, Waltham, MA, USA; Kit No. K0721), following the manufacturer’s instructions. Extracted DNA was stored at −20 °C until further analysis. Two genetic markers were targeted for amplification: the second internal transcribed spacer (ITS2) region and the cytochrome c oxidase subunit I (COXI) gene. The ITS2 region was amplified using previously described primers: ITS2-F (5′-TGTGTCGATGAAGAGCGCAG-3′) and ITS2-R (5′-TGGTTAGTTTCTTTTCCTCCGC-3′) [13]. For the COXI gene, primers COXI-F and COXI-R (5′-CATCATATGTTTATGGTGGGTTT-3′, 5′-GCAACCACAAACCATGTATCA-3′) were designed using NCBI’s Primer-BLAST tool. (https://www.ncbi.nlm.nih.gov/tools/primer-blast/, accessed on 10 July 2024).

PCR amplifications were carried out in 25 μL reaction volumes containing 100 ng of template DNA, 1× PCR buffer, 1 pmol of each primer, 2 mM MgSO_4_, 0.2 mM dNTPs, and 2.5 units of Taq polymerase (abm, Richmond, BC, Canada). A negative control was included in each PCR run using nuclease-free water in place of DNA.

The PCR cycling conditions for ITS2 amplification included initial denaturation at 94 °C for 1 min; 35 cycles of 94 °C for 1 min, 53 °C for 90 s, and 72 °C for 1 min; and a final extension at 70 °C for 10 min and hold at 10 °C [13]. For the COXI gene of Paramphistomidae, the PCR protocol was initial denaturation at 94 °C for 5 min; 35 cycles of 95 °C for 1 min, 51 °C for 30 s, and 72 °C for 1 min; and a final extension at 72 °C for 10 min and hold at 10 °C. The same protocol was used for the *Faciola* COXI amplification, except for the annealing temperature, which was adjusted to 55 °C. 

DNA and PCR product integrity were assessed using 0.8% and 1.5% agarose gel electrophoresis, respectively, run at 100 V and 90 mA for 60 min using an EC 1000-90 power supply (Thermo Electron, Waltham, MA, USA). Gels were stained with ethidium bromide and visualized under UV light using a Dual Wavelength UV transilluminator (Cleaver Scientific, Rugby, UK; SKU: CSLUV-TSDUOL). Gel images were subsequently digitized for documentation.

### 2.3. Sequence and Phylogenetic Analysis

PCR products were sequenced in both forward and reverse directions using the Sanger method with the same primers described previously. Sequencing was carried out at the Linkage and Research Laboratory of Universidad de Las Américas (UDLA). The resulting chromatograms were edited and assembled into consensus sequences using CodonCode Aligner (version 1.6.1). These sequences were then compared to those in GenBank using the BLAST tool, selecting matches with an identity greater than 90%.

To root the phylogenetic tree, *Taenia solium* sequences (accession numbers AF372569.1 and S69013.1) were included as outgroup taxa. Additional sequences used for comparison are listed in Table 1. Multiple sequence alignments were performed in MEGA11 (version 0.1) using the MUSCLE algorithm and refined with GBLOCKS 0.91b to remove poorly aligned regions.

Phylogenetic analysis was conducted using the Maximum Likelihood (ML) method with 1000 bootstrap replicates, implemented through the RAxML tool on the CIPRES v3.3 platform. The resulting tree was visualized and edited using FigTree v1.4.4.

## 3. Results

### 3.1. Specimens Studied

**Figure 2:**
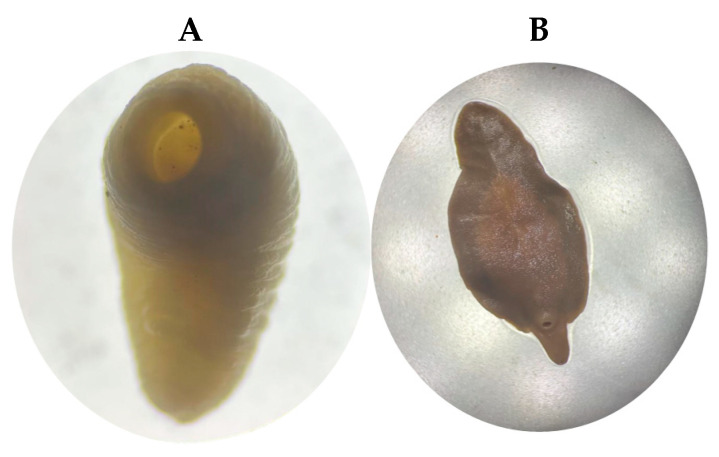
In Panel (**A**), a photograph shows a *Cotylophoron cotylophorum* specimen collected from the ruminal mucosa of a female bovine during postmortem examination. In Panel (**B**), *Fasciola hepatica* specimens recovered from the bile ducts of a slaughtered bovine are shown.

During the necropsy of a female bovine that likely died from paratuberculosis in Santo Domingo, Ecuador, several amphistome trematodes, likely of the family Paramphistomidae (n = 12), were found firmly attached to the ruminal papillae. These parasites exhibited a pinkish–reddish coloration, measured approximately 5–10 mm in length, and displayed a slight ventral curvature (Figure 2A). Also, in a veterinary examination at the Ambato slaughterhouse, a distinctive leaf- or lance-shaped, fleshy flatworm (platyhelminthe) was identified in the bile ducts (n = 9). It was approximately 2 to 3 cm long by 1.5 cm wide and was reddish-brown (Figure 2B). Because distinguishing Paramphistomidae (rumen flukes) is complicated, we decided to perform a molecular study.

### 3.2. PCR Detection

Specific ~500 bp amplicons were obtained for the ITS2 region, and ~266 bp fragments for the COXI gene, from both Paramphistomidae and Fasciolidae specimens, respectively. Tree sequencing was performed in both directions, and the resulting reads were aligned to generate consensus sequences.

### 3.3. Phylogenetic Analysis

**Figure 3:**
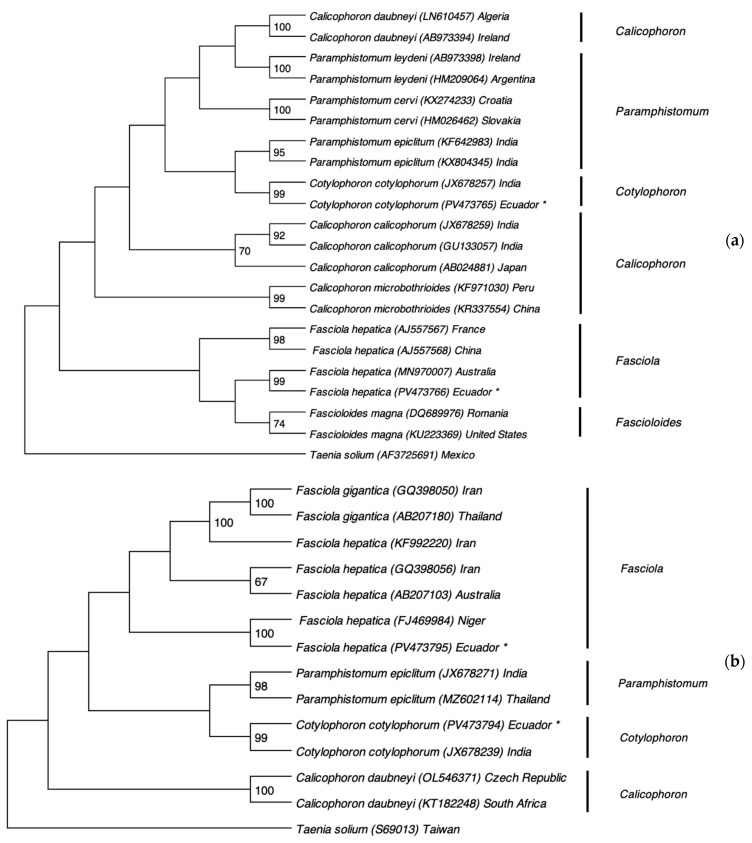
Phylogenetic analysis of ruminal and hepatic flukes based on the ITS2 region and COXI gene using the Maximum Likelihood method with 1000 bootstrap replicates. (**a**) Phylogenetic tree constructed from ITS2 sequences of trematodes. (**b**) Phylogenetic tree based on COXI gene sequences. Newly obtained sequences from this study are marked with an asterisk (*).

Figure 3 presents the phylogenetic analysis based on ITS2 and COXI gene sequences. The trematode species identified in this study clustered closely with reference sequences from the GenBank database, confirming their taxonomic identities. The ITS2 and COXI sequences of the Paramphistomidae specimen showed a 99% similarity with the Indian strain of *Cotylophoron cotylophorum* (accession numbers JX678257.1 and JX678239.1, respectively). Likewise, the ITS2 and COXI sequences of *Fasciola hepatica* matched with 99% and 100% similarity to the Australian (MN970007.1) and Nigerian (FJ469984.1) strains, respectively. The sequences of the fluke species of this study were deposited in GenBank under the following accession numbers: PV473765 (ITS2 of *Cotylophoron cotylophorum*), PV473794 (COXI of *Cotylophoron cotylophorum*), PV473766 (ITS2 of *Fasciola hepatica*), PV473795 (COXI of *Fasciola hepatica*).

Table 1 and Table 2 display the genotypic differences in the ITS2 region between our isolates and reference sequences, specifically at nucleotide position 147 for *Cotylophoron* spp. and position 212 for *Fasciola* spp.

## 4. Discussion

This study analyzed the ITS2 and COXI gene sequences of *rumen and liver flukes* collected in the Santo Domingo province, Ecuador. The sequences of the rumen flukes exhibited a 99% genetic similarity to an Indian strain of *C. cotylophorum*, indicating a close evolutionary relationship. This is the first confirmed record of this parasite in Ecuador. The ITS2 and COXI sequences of *Fasciola* spp. showed 99% and 100% similarity with the Australian and Nigerian strains of *F. hepatica*, respectively. In the ITS2 region, heterozygous positions were identified at nucleotide 147 in *C. cotylophorum* and at position 212 in *F. hepatica*, including an A/G overlap in the latter, supporting their diploid genome structure (Table 1 and Table 2). Both *F. hepatica* and *F. gigantica* are known to be diploid (2n = 20), although triploid forms (2n = 30) have also been reported in Japan [14,15,16]. Parthenogenetic diploid lineages may originate from hybridization between sexually reproducing populations, resulting in individuals with mixed nuclear genomes from both species [17,18]

Comparison of the COXI and ITS2 sequences from both parasites revealed the formation of two distinct clades: *Cotylophoron* was clearly separated from *Fasciola* spp., while *Taenia solium* clustered independently as an outgroup. These results confirm the evolutionary divergence of these trematodes while supporting their common ancestral origin. The phylogenetic trees also demonstrated a close genetic relationship between the Ecuadorian trematode genotypes and strains from other countries. Specifically, the ITS2 and COXI sequences of *C. cotylophorum* showed 99% similarity to Indian strains (JX678257.1 and JX678239.1), suggesting a possible Indian origin. This could be linked to the historical introduction of zebu cattle (*Bos taurus indicus*) from India to the Americas in the late 19th century [19], or to the importation of domestic water buffalo (*Bubalus bubalis*), a species native to Asia [20].

In the case of *Fasciola hepatica*, the ITS2 and COXI sequences showed 99% and 100% similarity with Australian (MN970007.1) and Nigerian (FJ469984.1) strains, respectively. It is likely that *F. hepatica* was introduced to Ecuador via infected domestic animals during colonization. Human-mediated introduction is also plausible, as this parasite is zoonotic and can infect people [6]. COXI-based phylogeny also distinguished liver flukes and rumen flukes into separate clades, which is consistent with the distribution of these parasites in the host. In this study, only *F. hepatica* was identified, with no evidence of *F. gigantica* or parthenogenetic forms as found in sheep in Ecuador [21]. All these species can be found in snail hosts in Ecuador, like Galba primos, G. coupe, G. cubensis, and Pseudosuccinea columella—genetically related to G. truncatula [7,22,23,24,25,26,27].

PCR and sequencing effectively identified *C. cotylophorum* and *F. hepatica* by amplifying ribosomal (ITS2, ~500 bp) and mitochondrial (COXI, ~266 bp) regions. ITS2 primers have been validated across multiple life stages (eggs, juveniles, adults) of various *Paramphistomid* genera [28], while the COXI region is widely recommended for genetic-diversity studies due to its high resolution [10,11,12]. Replicate sequencing of each sample in this study ensured the accuracy of the consensus sequences.

While COXI sequences were identical (100% similarity), ITS2 consensus sequences showed minor variation: a single nucleotide polymorphism (SNP) at position 147 in *C. cotylophorum* (1/483 bp; 0.21%) and at position 212 in F. hepatica (1/562 bp; 0.17%). These differences reflect distinct evolutionary pressures: non-coding regions such as ITS2 evolve more rapidly in the absence of functional constraints, whereas coding regions like COXI are under stronger purifying selection due to their role in essential proteins [29]. Moreover, ITS2 variation does not necessarily correlate with host species or geographic origin [30] because concerted evolution maintains uniformity among ribosomal repeats within a species, resulting in low intraspecific variability [31].

Both parasites infect ruminants, including the cattle from which our specimens were collected. They also share similar life cycles, using overlapping definitive and intermediate hosts [32,33,34,35,36], suggesting a potential common route of introduction to South America. However, phylogenetic analysis contradicts this: *C. cotylophorum* clusters with Indian isolates, whereas *F. hepatica* aligns with Nigerian (COXI) or Australian (ITS2) strains.

Determining precise origins is challenging because comparisons rely solely on sequences available in NCBI. Nevertheless, our data suggest that *C. cotylophorum* likely originated from India, while *F. hepatica* may trace back to Australia, Nigeria, or—even more plausibly—North America. Historical records show that during the colonial period, *F. hepatica* was introduced via livestock imports from Africa across the Pacific or via migrations from neighboring Colombia and Peru. In the post-colonial era, further introductions occurred from Europe, North America, and Central America [37]. The ITS2 sequences from Romania, Australia, North America, and Ecuador share a common ancestor (Figure 3a), supporting North America as the most probable source for Ecuador’s *F. hepatica* population.

Precise molecular identification of these trematodes has dual value: it improves understanding of parasite biology—especially life cycles and host interactions—and informs veterinary and public health interventions. It also sheds light on the geographic origins of these parasites in Ecuador, guiding policies on livestock movement and helping trace the introduction and spread of trematodes in the region.

## 5. Conclusions

This study presents the first report of the presence and molecular identification of *Cotylophoron cotylophorum* in Ecuador. This conclusion is based on DNA sequencing of the ITS2 and COXI regions, which showed the highest homology with *C. cotylophorum* among the Paramphistomidae family. In contrast, the ITS2 and COXI sequences from the Fasciolidae family, particularly *Fasciola hepatica* from Ecuador, exhibited close similarity to strains from Australia and Nigeria, respectively.

Phylogenetic analysis revealed that although these trematodes are evolutionarily related, they form two clearly distinct clades corresponding to the Paramphistomidae and *Fasciolidae* families. Additionally, single nucleotide polymorphisms (SNPs) were identified in the ITS2 consensus sequences of both parasites, supporting the diploid nature of these species. These findings further highlight ITS2 as an effective genetic marker for characterizing platyhelminth species, regardless of phenotype or geographic origin.

The introduction of these flukes into Ecuador is likely linked to historical imports of livestock, particularly *Bos indicus*, which was favored for its adaptability to tropical climates.

Molecular identification of parasites offers numerous advantages, including accurate species recognition—even among morphologically similar or cryptic taxa—and clarification of life cycles and transmission routes. It enables early and sensitive detection of infections, genetic differentiation of strains, and the identification of drug resistance markers. Additionally, it supports phylogenetic and evolutionary analyses, monitors parasite introduction and spread, aids in the development of diagnostic tools, and contributes to biodiversity conservation and public health surveillance. These tools are therefore essential for guiding control strategies and research on trematodes in Ecuador and globally.

## Figures and Tables

**Table 1:** The nucleotide sequences of the ITS2 region of Paramphistomids, downloaded from the NCBI and sequenced in this study, show the variation at nucleotide 147.

Access Codes ITS2	Species	Location	Variation Position (147)
PV473765	*Cotylophoron cotylophorum*	Ecuador	Y
JX678257.1	*Cotylophoron cotylophorum*	India	C
LN610457.1	*Calicophoron daubneyi*	Algeria	C
AB973394.1	*Calicophoron daubneyi*	Ireland	C
GU133057.1	*Calicophoron calicophorum*	India	C
JX678259.1	*Calicophoron calicophorum*	India	C
AB042188.1	*Calicophoron calicophorum*	Japan	C
KR337554.1	*Calicophoron microbothrioides*	China	C
KF791030.1	*Calicophoron microbothrioides*	Peru	C
HM209064.1	*Paramphistomum leydeni*	Argentina	C
AB973398.1	*Paramphistomum leydeni*	Ireland	C
KX274233.1	*Paramphistomum cervi*	Croatia	C
HM026462.1	*Paramphistomum cervi*	Slovakia	C
KF642983.1	*Paramphistomum epiclitum*	India	C
KX840345.1	*Paramphistomum epiclitum*	India	C

**Table 2:** The nucleotide sequences of the ITS2 region of Fasciola, downloaded from the NCBI and sequenced in this study, show the variation at nucleotide 212.

Access Codes ITS2	Species	Location	Variation Position (212)
PV473766	*Fasciola hepatica*	Ecuador	Y
MN970007.1	*Fasciola hepatica*	Australia	C
AJ557567.1	*Fasciola hepatica*	France	C
AJ557568.1	*Fasciola hepatica*	China	C
OQ689976.1	*Fascioloides magna*	Romania	C
KU232369.1	*Fascioloides magna*	United States	C

## Data Availability

The raw data supporting the conclusions of this article are available upon request. The corresponding authors will provide the data to any interested researcher upon formal request.
